# New Insights on Rotenone Resistance of Complex I Induced by the m.11778G>A/*MT-ND4* Mutation Associated with Leber’s Hereditary Optic Neuropathy

**DOI:** 10.3390/molecules27041341

**Published:** 2022-02-16

**Authors:** Francesco Musiani, Laura Rigobello, Luisa Iommarini, Valerio Carelli, Mauro Degli Esposti, Anna Maria Ghelli

**Affiliations:** 1 Dipartimento di Farmacia e Biotecnologie (FABIT), Università di Bologna, I-40126 Bologna, Italy; francesco.musiani@unibo.it (F.M.); laura.rigobello@studio.unibo.it (L.R.); luisa.iommarini2@unibo.it (L.I.); 2 Dipartimento di Scienze Biomediche e Neuromotorie (DIBINEM), Università di Bologna, I-40100 Bologna, Italy; 3 IRCCS Istituto delle Scienze Neurologiche di Bologna, Programma di Neurogenetica, I-40139 Bologna, Italy; 4 Center for Genomic Sciences, Universidad Nacional Autónoma de México (UNAM), Cuernavaca 62210, Mexico; mauro1italia@gmail.com

**Keywords:** complex I, LHON, mtDNA mutations, rotenone

## Abstract

The finding that the most common mitochondrial DNA mutation m.11778G>A/*MT-ND4* (p.R340H) associated with Leber’s hereditary optic neuropathy (LHON) induces rotenone resistance has produced a long-standing debate, because it contrasts structural evidence showing that the ND4 subunit is far away from the quinone-reaction site in complex I, where rotenone acts. However, recent cryo-electron microscopy data revealed that rotenone also binds to the ND4 subunit. We investigated the possible structural modifications induced by the LHON mutation and found that its amino acid replacement would disrupt a possible hydrogen bond between native R340 and Q139 in ND4, thereby destabilizing rotenone binding. Our analysis thus explains rotenone resistance in LHON patients as a biochemical signature of its pathogenic effect on complex I.

## 1. Introduction

**Figure 1:**
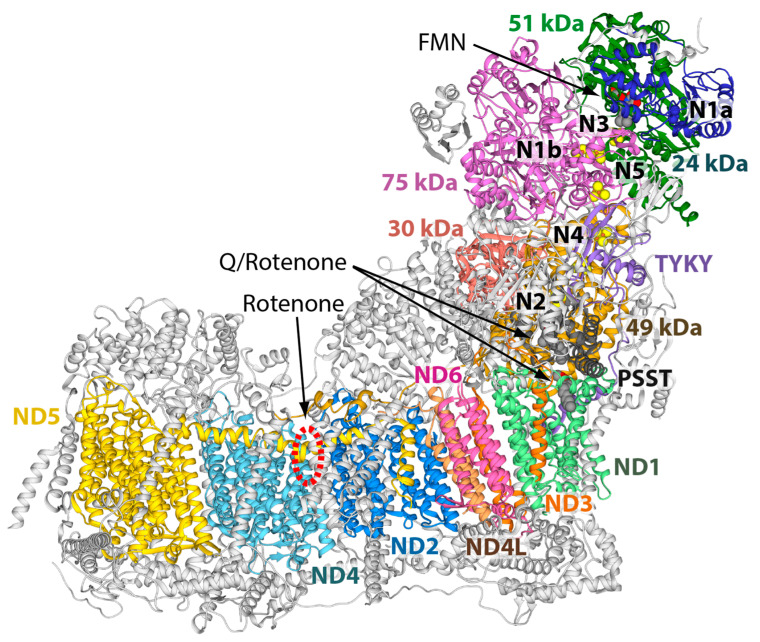
Structure of ovine respiratory CI (PDB ID 6ZKC) in ribbon representation [11]. Core subunits are colored, while supernumerary ones are in grey. The cofactors are reported as spheres colored according to the atom type, as well as the decyl-quinone molecules found in the structure. EPR visible FeS clusters are named according to [1,16]. The rotenone binding sites are also indicated: two sites correspond to the decyl-quinone sites, while the third site is located in the P-module in the ND4 subunit (dashed red circle).

In the last 10 years, our knowledge of the respiratory complex I (CI) structure has dramatically improved thanks to a wealth of atomic data obtained from both X-ray crystallography and cryo-electron microscopy (cryo-EM) technologies [1,2]. These different approaches have provided valuable information to clarify the mechanism that couples NADH: ubiquinone oxidoreduction to proton pumping. The overall structure of CI is conserved (Figure 1) and consists of a peripheral and a membrane arm containing 14 conserved catalytic “core subunits” [3]. In addition, depending on the organism, there is a variable number of “supernumerary” subunits that contribute to the regulation, stability, and assembly of this enzyme [4,5]. CI can be divided into three main functional modules: N- and Q-modules located in the peripheral arm and the P-module corresponds to the membrane arm [6]. The N-module contains one noncovalently bound flavin mononucleotide (FMN) and six [4Fe4S] and two [2Fe2S] iron–sulfur (FeS) clusters as primary electron acceptors, whereas the Q-module encloses the last [4Fe4S] cluster (N2) and the quinone binging site in which ubiquinone (Q) is reduced [6]. Lastly, the P-module contains proton pumping devices consisting of ND1, ND6, and ND4L subunits forming together the so-called E-channel, and the antiporters subunits ND2, ND4, and ND5 [6]. Combination of structural kinetic, spectroscopic, and computational analysis led to the proposal of several molecular mechanisms to explain the coupling between the energy released by the electron transport from NADH to ubiquinone and proton pumping (reviewed in [1,2]). Specific inhibitors of CI, such as plant-derived rotenone, a classical inhibitor of mitochondrial respiration, have been instrumental for several of these proposals [7,8,9]. Nowadays, CI is co-crystalized with substrate-like inhibitors, such as piericidin A, 2-decyl-4-quinazolinyl amine, acetogenins, and rotenone itself [10,11,12,13,14]. The picture emerging from structural data is that these CI inhibitors bind to two different sites along the wormhole-like cavity accommodating the Q substrate in its various redox states within the enzyme complex (reviewed in [1] and [10,11]). Such a picture would explain early evidence for two rotenone and piericidin A binding sites in mammalian CI and their mutual exclusivity, but mixed antagonist action versus Q substrates [7,15].

Very recently, Kampjut and Sazanov reported cryo-EM structures of ovine CI in the presence or absence of both substrates (NADH and decyl-ubiquinone) and rotenone, showing that the latter also binds to a third site in the ND4 subunit (Figure 1) [11].

This recent structural information can be correlated with the finding that we reported in 1994—that human mitochondria harboring a pathogenic mutation in the mitochondrially encoded ND4 subunit (*MT-ND4*) displays resistance to rotenone [17]. In particular, the mitochondrial DNA (mtDNA) primary mutation, m.11778G>A/*MT-ND4* (p.R340H), pathogenic for Leber’s hereditary optic neuropathy (LHON), induced a reduction in rotenone sensitivity to its inhibitory effect in vitro [17]. However, for a long time, it has been difficult to explain such a reduced sensitivity to rotenone inhibition implying reduced binding to the complex, since the ND4 subunit is located far away from the Q-site, where rotenone and other Q-antagonist inhibitors predominantly bind to CI. We can now explain the rotenone resistance found in LHON patients carrying the most frequent LHON mutation p.R340H on the basis of the new atomistic resolution data from the cryo-EM structure of ovine CI bound to rotenone [11].

## 2. Results and Discussion

In two recent cryo-EM ovine structures of CI bound to rotenone [11] (PDB ID 6ZKM and 6ZKN), an additional binding site for the inhibitor was found. In particular, this third site for rotenone lies within the ND4 subunit (Figure 1). ND4 is a highly conserved transmembrane protein with 35% conserved amino acids in Eukaryota, 63% in Vertebrata, and more than 77% in Mammalia (see Table 1).

**Figure 2:**
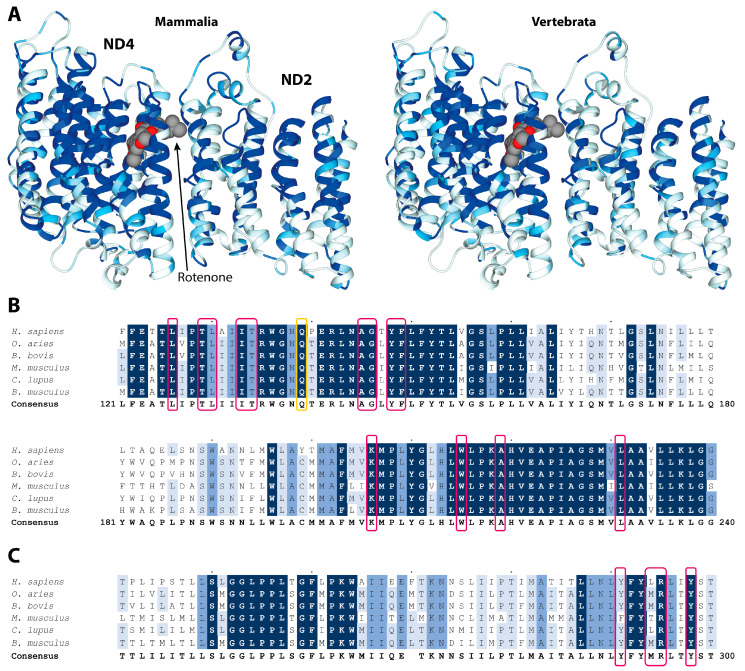
(**A**) Residue conservation mapped on the structure of rotenone bound ND4 and ND2 subunits (PDB ID 6ZKM) for Mammalia (left panels) and Vertebrata (right panels). The ribbon color goes from white (0% conservation) to light blue (70%), medium blue (90%), and dark blue (100%). The rotenone is reported in a space-fill representation, colored according to the atom type. (**B**,**C**) Multiple alignment and conservation analyses of ND4 and ND2 protein regions binding rotenone. Alignments of protein sequences from mammals, including *Homo sapiens* used as the reference sequence, are reported. Different shading corresponds to increasing conservation levels: amino acid conservation between 70% and 90% are highlighted in light blue, amino acid conservation between 90% and 99% are highlighted in medium blue, and invariant positions (100% conservation) are highlighted in dark blue. Alignment gaps are indicated by a dash (-). Amino acids involved in rotenone binding are boxed in pink, while those undergoing conformational changes in LHON mutant are boxed in yellow.

The most conserved residues in the 14 transmembrane helices can be seen in Figure 2A,B and in Supplementary Figure S1, forming a hydrophilic axis along the middle plane of the mitochondrial membrane, which is critical for the proton pumping mechanism, as previously reported [12].

Rotenone is buried within helices TM5, TM6, and TM7 of ND4, and forms extensive interactions with 13 highly conserved residues (Figure 2B and Supplementary Figure S1). Indeed, 10 out of these 13 residues are invariant in vertebrates, highlighting their functional roles. In particular, W215 and L231 are invariant in eukaryotes, while K206, which is critically involved in forming the proton channel contributed by the ND4 subunit, is almost invariant [11]. Rotenone also forms multiple interactions with four amino acids belonging to the ND2 subunit that highly conserve, especially in vertebrates, such as R295 and Y298 (Figure 2C and Supplementary Figure S2).

**Figure 3:**
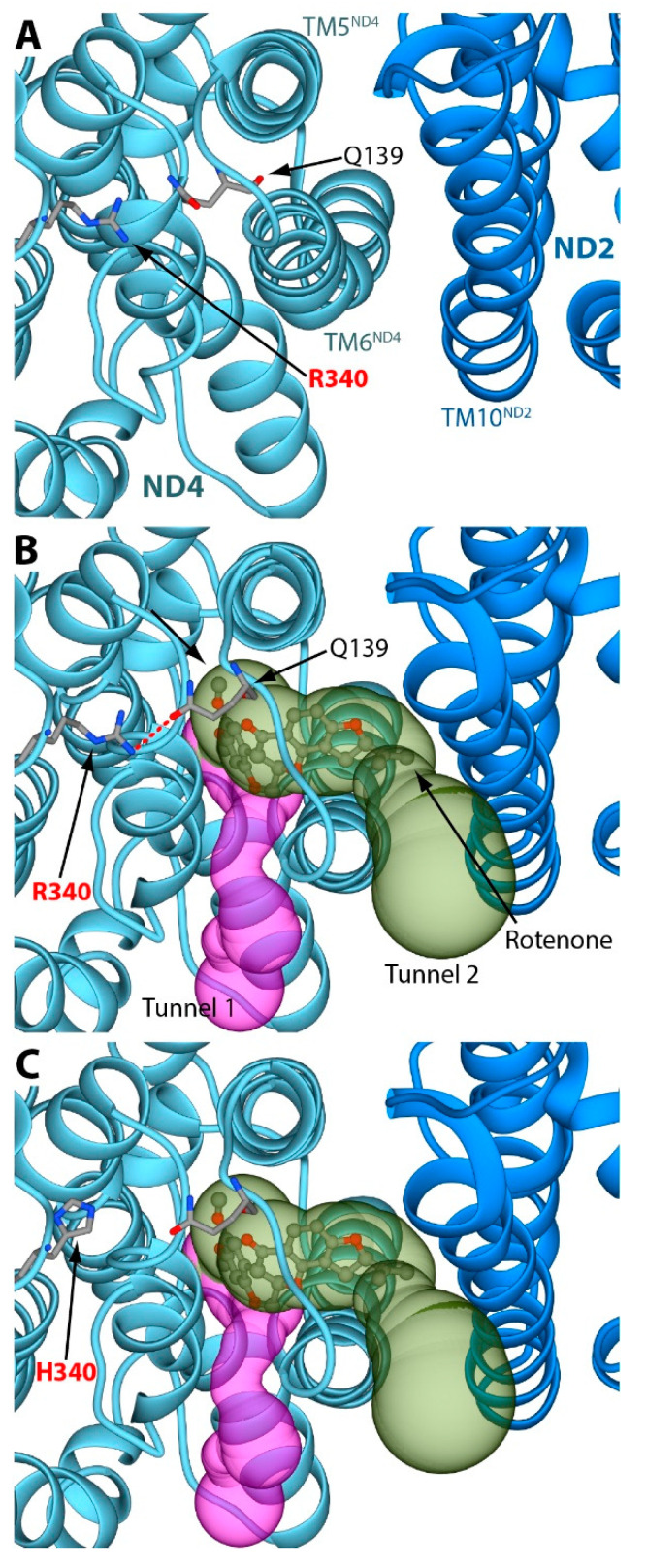
(**A**) Details of the rotenone binding site without the inhibitor (PDB ID 6ZKC). The ND4 and the ND2 subunits are reported as light blue and blue ribbons, respectively. The amino acids discussed in the text are reported as sticks, colored according to the atom type. (**B**) The same region of panel **A** in a CI structure bound to rotenone (PDB ID 6ZKM). Rotenone is shown as balls-and-sticks, colored according to the atom type. The two tunnels starting from the rotenone binding pocket are reported as purple and green surfaces. The H-bond between R340 and Q139 is highlighted using a red dashed line. (**C**) The same as panel **B**, but with R340 mutated in silico into a histidine residue. In all panels, the orientation of the protein is rotated by 90° around the horizontal axis, with respect to the orientation reported in Figure 1.

The analysis of the third rotenone binding pocket reveals some striking structural differences in ND4 and the closely associated ND2 after binding of the inhibitor (Figure 3A and Figure 3B, respectively).

The rotenone-binding pocket is located between helices TM5 and TM6 of ND4, where the inhibitor is able to block one of the proton translocation pathways of CI [11]. Specifically, rotenone induces a conformational change in ND4 helix TM6 and in ND2 helix TM10 [11]. Using the MOLEonline 2.5 software (https://mole.upol.cz/, accession date 27 July 2021), we observed that such conformational rearrangement produces a large cavity between ND4 helices TM5 and TM6, which is large enough to accommodate the bulky rotenone molecule (Figure 3B). The role of R340 in this rotenone binding site would appear to be elusive, since the residue lies on the loop connecting ND4 helices TM11 and TM12 that is not in direct contact with the inhibitor-binding pocket (Figure 3A,B, and [11]). However, a 180° rotation of the Q139 Cδ–Cγ bond, a mildly conserved residue in the proximity of R340, suggests the possible formation of a hydrogen bond between the R340 Nω1 atom and the Q139 Oε1 atom (Figure 3B). Q139 lies between some highly-conserved residues involved in the formation of the rotenone binding cavity (i.e., I133, T134, A145, and G146) [11]. Reproduction of the LHON mutation in silico shows that R340 replacement with histidine prevents the formation of any possible hydrogen bond with Q139 (Figure 3C). Therefore, we hypothesize that the modeled R340-Q139 hydrogen bond would be able to maintain the ND4 helix TM6 in a conformation distant from helix TM5, favoring the formation of the third rotenone-binding cavity. On the other hand, the absence of the R340–Q139 hydrogen bond can cause a larger conformational mobility of the ND4 helix TM6, reducing the probability of successful rotenone interactions. This would explain the observed mild resistance to rotenone (about three-fold on average) in mitochondria harboring the m.11778G>A/*MT-ND4* mutation [17,18]. This resistance is mild, likely because most of the rotenone-inhibitory effect depends on its binding into the Q-site cavity, but very specifically. Indeed, in the same platelet mitochondria, we demonstrated that the sensitivity to rolliniastatin-2, one of the most powerful Q-inhibitors of CI, was unaltered in LHON patients; moreover, the sensitivity of other CI inhibitors, such as stigmatellin and amytal, was also the same in the controls and patients’ mitochondria [17,18]. It was reported that CI shows rotenone resistance in its de-active form [19], but this situation could hardly apply to our biochemical assays of CI, which were performed after the incubation with NADH (i.e., with the equilibrium shifted towards the active form of CI [19]). This rules out the possibility that the observed resistance to rotenone may derive from a different proportion of the de-active form CI in controls and LHON patients [17,18]. Notably, the third binding site of rotenone in ND4 is close to the proton pumping machinery of CI, in which ND4 plays a critical role (Figure 1, cf. [11]). The structural change imposed by the LHON mutation p.R340H may thus reduce the energy conservation capacity of CI and determine its pathological role. In this view, rotenone inhibition, also reflecting its binding to the third site, becomes a biochemical tool to highlight the structural alteration underlying the mitochondrial dysfunction in LHON patients. Remarkably, our previous observation of rotenone resistance in platelets of LHON patients carrying the p.R340H change was instrumental in defining the pathogenic role of this variant. Indeed, we and others failed to document a significant and specific impairment on CI electron transfer activity [18,20], which was shown to be only slightly reduced [21,22]. Before this evidence, the pathogenicity of LHON p.R340H change was considered to arise only from a general reduction in NADH respiration [23]. Conversely, the less frequent LHON mutation m.3460G>A/*MT-ND1* (p.A52T) clearly affected the electron transfer to the quinone substrate, as well as the sensitivity to rotenone inhibition [18,20,21,22,24,25]. In this case, the biochemical alteration fitted well with the role of the ND1 subunit in binding Q and its antagonist inhibitors. Indeed, the ND1 subunit was later found to form the entrance of the Q wormhole-like site of CI [1]. Last, the third (and universally recognized as less severe) LHON mutation, m.14484T>C/MT-ND6 (p.M64V), also leaves the electron transfer substantially unaffected, while altering sensitivity of CI to myxothiazol and nonyl-benzoquinol, but not to rotenone [26]. Several years ago, we interpreted these changes in rotenone sensitivity as suggestive of an altered catalytic activity of CI leading a partial decrease of net energy production and a chronic increase of oxidative stress that in turn induced cell death [17,18,27,28,29,30]. Subsequent studies demonstrated that in LHON cells, CI-driven ATP synthesis was severely affected, reactive oxygen species (ROS) production was increased, and cells were prone to undergo apoptosis. Furthermore, in vivo results demonstrated a defective ATP synthesis in skeletal muscles and/or the brain using the ^31^P magnetic resonance spectroscopy in LHON patients [31,32].

In light of the current results, integrated with the previous biochemical phenotyping of the three CI LHON mutations affecting, respectively, the ND4, ND1, and ND6 subunits, we may envisage an overall scenario. The m.3460G>A/*MT-ND1* (p.A52T) mutation is the only one to affect the electron transfer, thus directly impinging on redox activity of CI, decreasing the quinol formation and downstream electron flow and proton pumping. This ultimately reflects the overall oxidative phosphorylation efficiency with increased ROS production, even if the relative weight of the two effects on the final mitochondrial dysfunction and disease pathogenesis remain unclear. As far as the other two mutations are concerned, the most frequent and severe m.11778G>A/*MT-ND4* and the less frequent and severe m.14484T>C/*MT-ND6* do not seem to consistently affect electron transfer to the quinone, while possibly affecting proton translocation across the inner mitochondrial membrane and, ultimately, energy conserving efficiency. Measuring final net ATP synthesis [33] and respiration [21], all three mutations clearly affect both outcomes. Again, the contribution of ROS production by each mutation is less clear, as electron flow remains relatively unaffected with the latter two. Overall, we faced two slightly different mechanisms of CI impairment, which may impact clinical phenotypes and therapy response, as for the currently used quinone analogue idebenone [30,34]. Besides the biochemical phenotypes, in fact, clinically, the m.11778G>A/*MT-ND4* mutation is considered the most severe, particularly regarding the lowest rate of spontaneous recovery of visual function in LHON-effected individuals [35], whereas the m.3460G>A/*MT-ND1* is somehow intermediate, and the m.14484T>C/*MT-ND6* is the less severe, with the highest rate of spontaneous visual recovery. Finally, another noticeable observation fitting the biochemical phenotype, not the clinical one, concerns the degree of association of LHON mutations with specific mtDNA haplotypes. Haplogroup J, characterized by population-specific variants affecting CI and complex III, has the highest association with the m.14484T>C/*MT-ND6* mutation, for which it is proposed that this mtDNA background is needed to express LHON. Conversely, the m.11778G>A/*MT-ND4* mutation has a weaker association with haplogroup J, and the m.3460G>A/*MT-ND1* mutation is not associated, suggesting that this latter mutation is sufficient by itself to produce enough disease penetrance [36,37].

## 3. Materials and Methods

### 3.1. Sequence Alignment and Conservation Determination

All of the reviewed protein sequences from ND1 to ND6 were downloaded from UniProtKB (https://www.uniprot.org/help/uniprotkb, accessed on 12 November2021). Fragment sequences were eliminated, and sequences were clustered in taxonomic groups, namely Eukaryota, Vertebrata, and Mammalia. The final number of aligned sequences was reported in Table 1. Sequences were aligned using Clustal Omega v:1.2.4 (https://www.ebi.ac.uk/Tools/msa/clustalo/, accessed on 12 November 2021) with the default parameters. The human sequences were set as reference sequences for numbering and consensus sequences and percentages of identity were calculated for each alignment by using Jalview v:2.11.1.4 [38]. Amino acids were considered as conserved if their sequence identities surpassed the conservation threshold of 70%.

### 3.2. Structural Analyses of the Rotenone Binding Site in the ND4 Subunit

The tunnels starting from the rotenone binding pocket have been calculated using the software MOLEonline [39] on the rotenone inhibited cryo-EM ovine structure, featuring the highest resolution (PDB ID 6ZKM) [11]. Amino acids interacting with rotenone have been identified considering those surrounding the inhibitor within a maximum distance of 4.0 Å. The reported H-bond was calculated using the *H-bonds* tool included in UCSF Chimera [40] and the R340H mutation was performed by using the *swapaa* tool from the same software. *H-bonds* uses atom types and geometric criteria to identify possible H-bonds, even in the absence of hydrogen atoms, as in the present structures. The geometric criteria are based on a survey of small-molecule crystal structures, as described by Mills and Dean [41]. *Swapaa* replaces amino acid sidechains using information from a rotamer library. A residue can be changed to a different sidechain conformation of the same type of amino acid or mutated into a different type. Rotamers are chosen automatically and optimized to reduce the clashes with the neighboring atoms, to optimize the largest possible number of H-bonds. In the mutant, the H340 side chain conformation was chosen from the Dunbrack library [42]. The latter uses a continuous probability density estimating for the non-rotameric degrees of freedom of amides, carboxylates, and aromatic sidechains. Subsequently, the sidechains were modeled as functions of the backbone dihedrals and rotamers of the remaining degrees of freedom [42].

### 3.3. Limitations

As with any modeling study, our model also has limitations. First of all, our considerations are based on relatively low-resolution structures, although they can be considered very good in the set of structures obtained so far by cryo-EM. Secondly, our hypothesis is based on a static structure, whereas a dynamic characterization by means of molecular dynamics calculations—which are beyond the scope of the present communication—could support our hypothesis, as could the cryo-EM structure of the R340H mutant.

## 4. Conclusions

The structural explanation that we present here for the previously puzzling effect of the m.11778G>A/*MT-ND4* mutation on complex I now offers new opportunities to refine the pathogenic mechanism of this and other mitochondrial DNA mutations. Moreover, the deeper structural knowledge of CI further contributes to our understanding of LHON, an elusive paradigm of mitochondrial diseases for which much effort has recently been placed to find a cure to avoid blindness.

## Figures and Tables

**Table 1:** Aligned protein sequences and conservation.

		ND1	ND2	ND3	ND4	ND4L	ND5	ND6
**Eukaryota**	Aligned sequences	159	166	158	105	331	109	129
	Average conservation (%)	70	60	61	59	69	55	44
	Conserved residues (%)	56	34	42	35	57	26	11
	Invariant residues (%)	2	1	2	3	1	4	0
**Vertebrata**	Aligned sequences	109	127	101	62	279	59	84
	Average conservation (%)	82	70	75	77	77	73	58
	Conserved residues (%)	77	51	62	63	64	56	29
	Invariant residues (%)	26	10	20	20	5	17	6
**Mammalia**	Aligned sequences	79	95	74	44	256	39	43
	Average conservation (%)	85	77	80	83	80	79	71
	Conserved residues (%)	79	62	70	77	69	68	55
	Invariant residues (%)	39	20	31	38	17	32	23

## Data Availability

Multiple alignments and the mutated CI structure are available upon request.

## Supplementary Materials

The following supporting information can be downloaded online, Figure S1: Multiple alignment and conservation analysis of ND4 protein sequences; Figure S2: Multiple alignment and conservation analysis of ND2 protein sequences.

## Sample Availability

The alignment and the models used in this work are available upon request from the authors.
