# Supplementary material for: New Insights on Rotenone Resistance of Complex I Induced by the m.11778G>A/MT-ND4 Mutation Associated with Leber’s Hereditary Optic Neuropathy
Source: Molecules. 2022 Feb 16;27(4):1341. doi: 10.3390/molecules27041341 (PMC8876992; doi:10.3390/molecules27041341)
Supplement: Supplementary file 1 [file molecules-27-01341-s001.zip › molecules-1541221-supplementary.pdf]

Musiani, F.; Rigobello, L.; Iommarini, L.; Carelli, V.; Degli Esposti, M. and Ghelli, A.M.

New Insights on Rotenone Resistance of Complex I Induced by the m.11778G>A/*MT-ND4* Mutation Associated with Leber's Hereditary Optic Neuropathy.

SUPPLEMENTARY MATERIAL

Supplementary Figure Legends

Figure S1. Multiple alignment and conservation analysis of ND4 protein sequences. Alignments of protein sequences from Eukaryota, Vertebrata and Mammalia, including *Homo sapiens* used as reference sequence, are reported. Different shading corresponds to increasing conservation levels: amino acid conservation between 70% and 90% are highlighted in light blue, amino acid conservation between 90% and 99% are highlighted in medium blue, and invariant positions (100% conservation) are highlighted in dark blue. Alignment gaps are indicated by a dash (-). Amino acids involved in rotenone binding are boxed in pink, while those undergoing conformational changes in LHON mutant (p.R340H) and its interacting residue (p.Q139) are boxed in yellow. Consensus sequence and secondary structure (PDB: 6ZKM) are also shown.

Figure S2. Multiple alignment and conservation analysis of ND2 protein sequences. Alignments of protein sequences from Eukaryota, Vertebrata and Mammalia, including *Homo sapiens* used as reference sequence, are reported. Different shading corresponds to increasing conservation levels: amino acid conservation between 70% and 90% are highlighted in light blue, amino acid conservation between 90% and 99% are highlighted in medium blue, and invariant positions (100% conservation) are highlighted in dark blue. Alignment gaps are indicated by a dash (-). Amino acids involved in rotenone binding are boxed in pink. Consensus sequence and secondary structure (PDB: 6ZKM) are also shown.

Figure S1

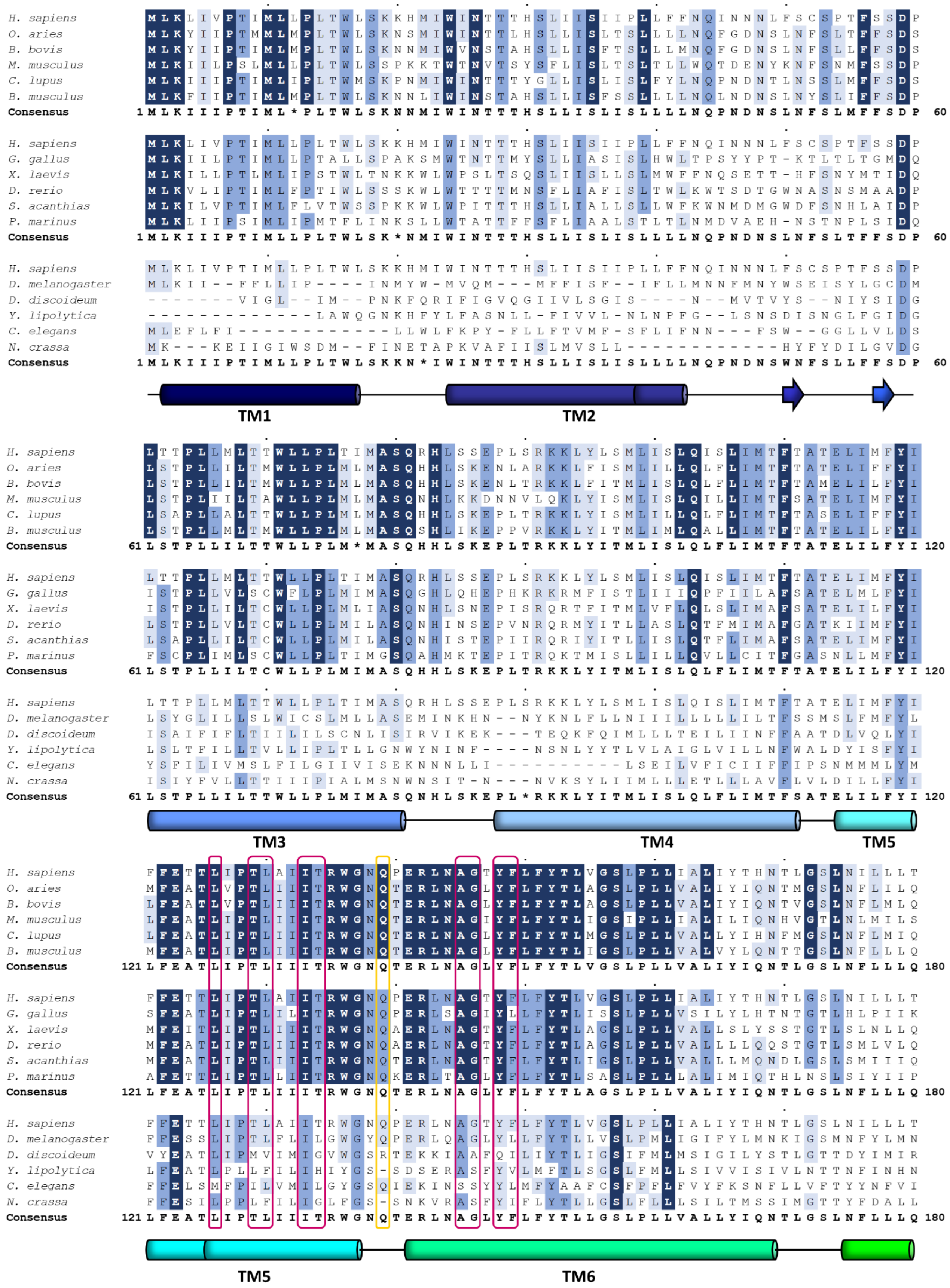
H. sapiens
O. aries
B. bovis
M. musculus
C. lupus
B. musculus
Consensus
1
60
H. sapiens
G. gallus
X. laevis
D. rerio
S. acanthias
P. marinus
Consensus
1
60
H. sapiens
D. melanogaster
D. discoideum
Y. lipolytica
C. elegans
N. crassa
Consensus
1
60
TM1
TM2
H. sapiens
O. aries
B. bovis
M. musculus
C. lupus
B. musculus
Consensus
61
120
H. sapiens
G. gallus
X. laevis
D. rerio
S. acanthias
P. marinus
Consensus
61
120
H. sapiens
D. melanogaster
D. discoideum
Y. lipolytica
C. elegans
N. crassa
Consensus
61
120
TM3
TM4
TM5
H. sapiens
O. aries
B. bovis
M. musculus
C. lupus
B. musculus
Consensus
121
180
H. sapiens
G. gallus
X. laevis
D. rerio
S. acanthias
P. marinus
Consensus
121
180
H. sapiens
D. melanogaster
D. discoideum
Y. lipolytica
C. elegans
N. crassa
Consensus
121
180
TM5
TM6

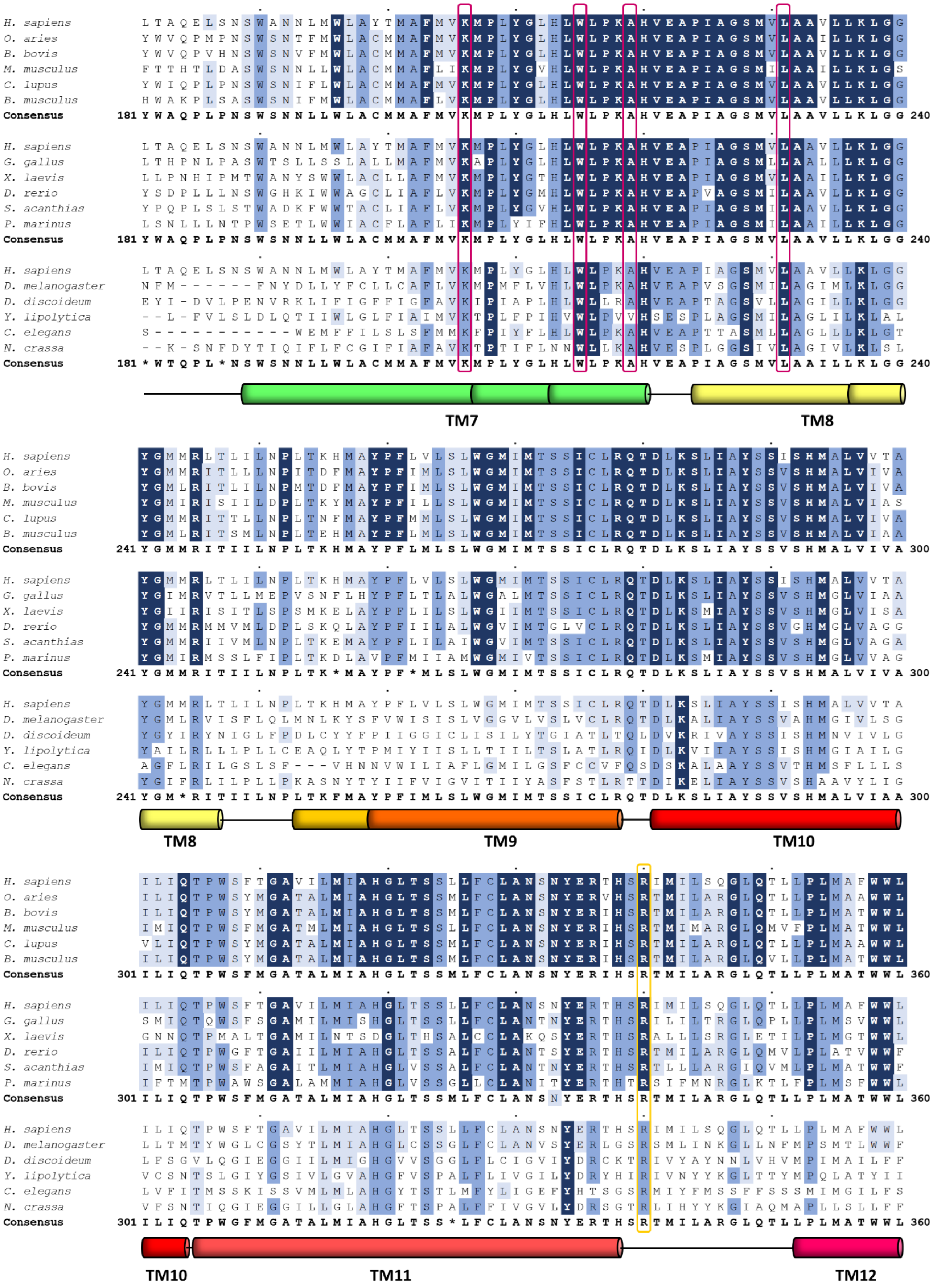

H. sapiens
O. aries
B. bovis
M. musculus
C. lupus
B. musculus
G. gallus
X. laevis
D. rerio
S. acanthias
P. marinus
D. melanogaster
D. discoideum
Y. lipolytica
C. elegans
N. crassa
Consensus
TM7
TM8
TM9
TM10
TM11
TM12

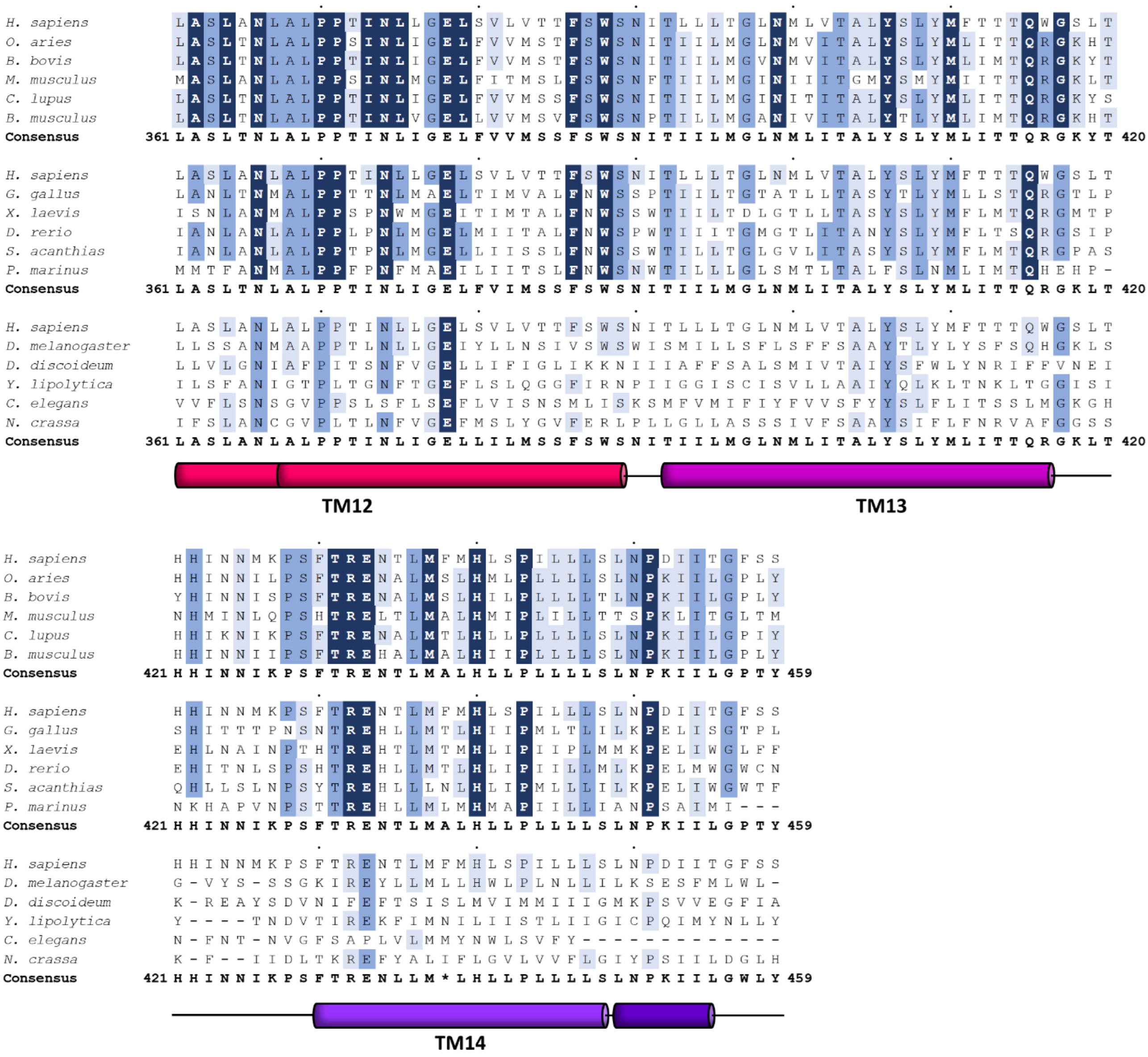
H. sapiens
O. aries
B. bovis
M. musculus
C. lupus
B. musculus
Consensus
361 LASLTNLALPPTINLIGELFVVMSSFSWSNITIILMGLNMLITALYSLYMLITTQRGKYT 420
H. sapiens
G. gallus
X. laevis
D. rerio
S. acanthias
P. marinus
Consensus
361 LASLTNLALPPTINLIGELFVIMSSFSWSNITIILMGLNMLITALYSLYMLITTQRGKLT 420
H. sapiens
D. melanogaster
D. discoideum
Y. lipolytica
C. elegans
N. crassa
Consensus
361 LASLANLALPPTINLIGELLILMSSFSWSNITIILMGLNMLITALYSLYMLITTQRGKLT 420
TM12
TM13
H. sapiens
O. aries
B. bovis
M. musculus
C. lupus
B. musculus
Consensus
421 HHINNIKPSFTRENTLMALHLLPLLLLSLNPKIILGPTY 459
H. sapiens
G. gallus
X. laevis
D. rerio
S. acanthias
P. marinus
Consensus
421 HHINNIKPSFTRENTLMALHLLPLLLLSLNPKIILGPTY 459
H. sapiens
D. melanogaster
D. discoideum
Y. lipolytica
C. elegans
N. crassa
Consensus
421 HHINNIKPSFTRENLLM*LHLLPLLLLSLNPKIILGWLY 459
TM14

Figure S2

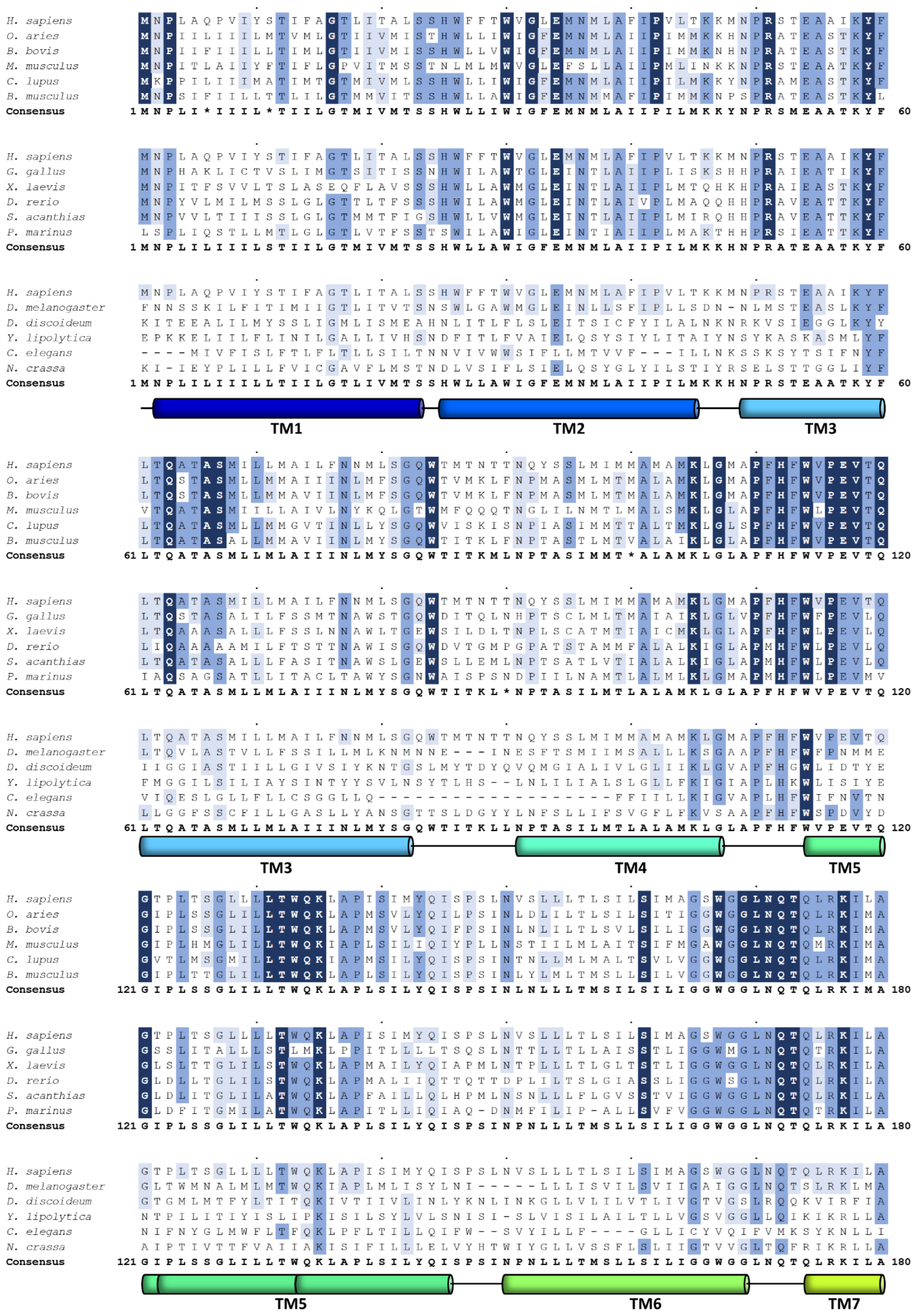
H. sapiens
O. aries
B. bovis
M. musculus
C. lupus
B. musculus
G. gallus
X. laevis
D. rerio
S. acanthias
P. marinus
D. melanogaster
D. discoideum
Y. lipolytica
C. elegans
N. crassa
Consensus
TM1
TM2
TM3
TM4
TM5
TM6
TM7

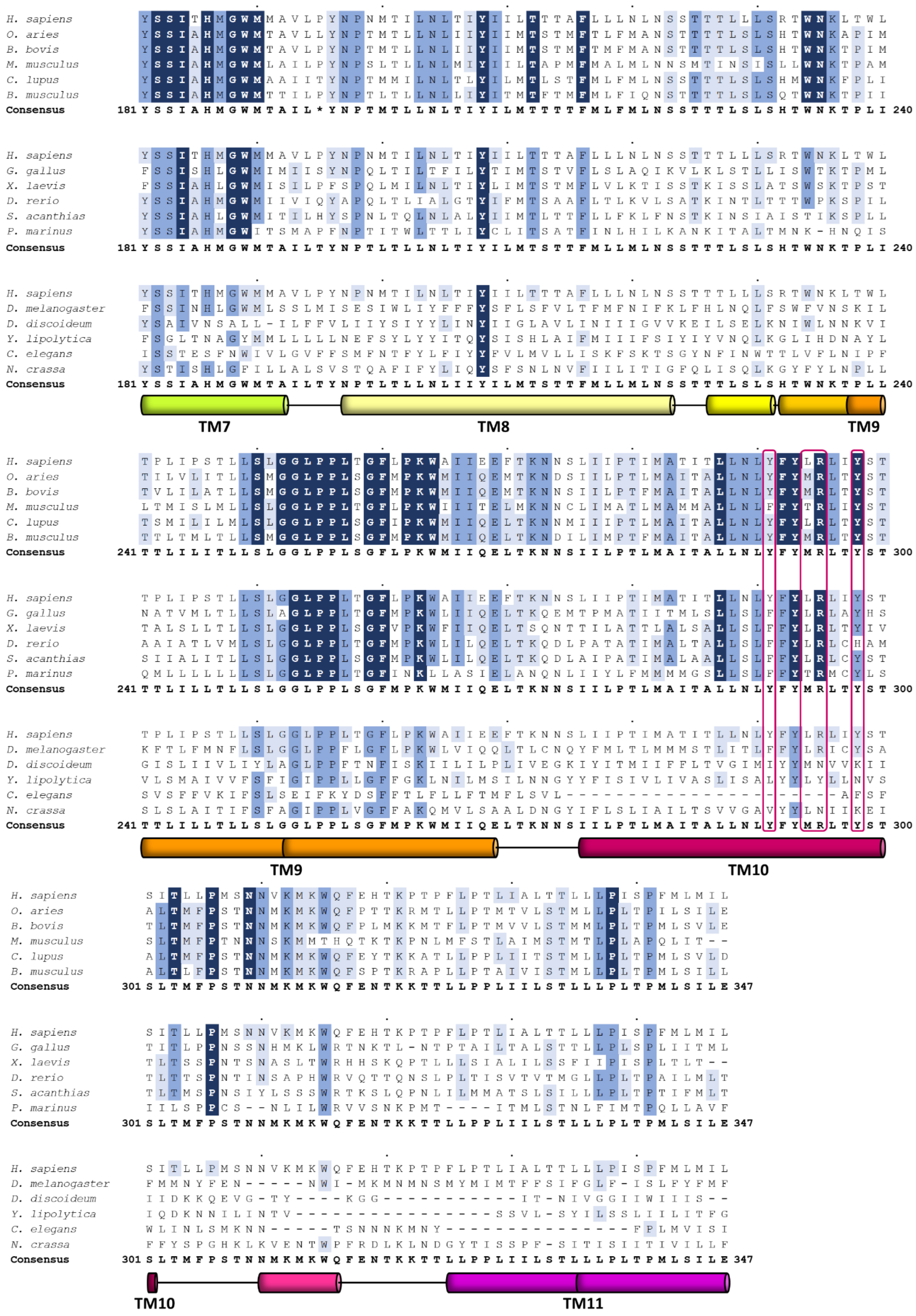
H. sapiens
O. aries
B. bovis
M. musculus
C. lupus
B. musculus
G. gallus
X. laevis
D. rerio
S. acanthias
P. marinus
D. melanogaster
D. discoideum
Y. lipolytica
C. elegans
N. crassa
Consensus
TM7
TM8
TM9
TM10
TM11
